# Expanding our Understanding of the Seaweed Holobiont: RNA Viruses of the Red Alga *Delisea pulchra*

**DOI:** 10.3389/fmicb.2015.01489

**Published:** 2016-01-08

**Authors:** Tim Lachnit, Torsten Thomas, Peter Steinberg

**Affiliations:** ^1^Centre for Marine Bio-Innovation, University of New South Wales, SydneyNSW, Australia; ^2^Zoological Institute, Christian-Albrechts-University KielKiel, Germany; ^3^School for Biotechnology and Biomolecular Science, University of New South Wales, SydneyNSW, Australia; ^4^School of Biological, Earth and Environmental Sciences, University of New South Wales, SydneyNSW, Australia; ^5^Sydney Institute of Marine Science, MosmanNSW, Australia

**Keywords:** viromes, disease, pathogen, seaweed, alga, fungi, macroalga, metaorganism

## Abstract

Marine seaweeds are holobionts comprised of the macroalgal hosts and their associated microbiota. While the composition of the bacterial component of seaweed microbiomes is increasingly studied, almost nothing is known about the presence, diversity and composition of viruses in macroalgae *in situ*. In this study, we characterize for the first time the viruses associated with a red macroalga, *Delisea pulchra.* Using transmission electron microscopy we identified diverse morphotypes of virus-like particles in *D. pulchra* ranging from icosahedral to bacilliform to coiled pleomorphic as well as bacteriophages. Virome sequencing revealed the presence of a diverse group of dsRNA viruses affiliated to the genus *Totivirus*, known to infect plant pathogenic fungi. We further identified a ssRNA virus belonging to the order *Picornavirales* with a close phylogenetic relationship to a pathogenic virus infecting marine diatoms. The results of this study shed light on a so far neglected part of the seaweed holobiont, and suggest that some of the identified viruses may be possible pathogens for a host that is already known to be significantly impacted by bacterial infections.

## Introduction

Marine seaweeds are colonized by diverse microbial communities (Lachnit et al., 2009, 2011, 2013; Bengtsson et al., 2012; Marzinelli et al., 2015), which interact with their hosts in both positive and negative ways (Matsuo et al., 2005; Fernandes et al., 2011; Campbell et al., 2014). As with other eukaryotes, seaweeds and microorganisms can be considered a “holobiont,” an intimately interacting association of microorganisms and their host (Egan et al., 2013). The relationship between the host and its microbiome can be fundamental to the functioning of the host, and when disturbed can result in disease (Case et al., 2011; Willing et al., 2011). This is particularly relevant to marine systems, where diseases can impact whole populations [e.g., seagrass wasting disease (Godet et al., 2008), coral or algal bleaching (Rosenberg et al., 2007; Campbell et al., 2011) or sea star wasting disease (Hewson et al., 2014)], and for which there is evidence for an increasing frequency or intensity of diseases (Harvell et al., 1999). This later observation may be due to increased environmental stress, such as temperature or eutrophication, which are potentially important factors that destabilize the holobiont and make it more susceptible to pathogen infection (Largo et al., 1995). Knowing all the association partners is the first step to understanding the health and functional outcomes for marine holobionts, and to the potential consequences of disease in these systems.

Seaweeds are the dominant habitat-forming organisms on temperate rocky reefs, comparable in importance to corals in the tropics (Graham, 2004). In recent years there have been a number of reports of diseases having significant impacts on seaweed populations and communities (Largo et al., 1995; Campbell et al., 2011, 2014). However, in general little is known about seaweed diseases in natural environments and most research done to date on the seaweed holobiont has focused on the identification and composition of surface-associated bacteria. Most of the causative agents identified are thus pathogenic bacteria, or, in a few instances, fungi (Gachon et al., 2010; Egan et al., 2014). The viral component of the seaweed holobiont is in contrast largely unknown.

Viruses are the most abundant entities in the marine environment (Suttle, 2005). They are able to control phytoplankton blooms (Suttle et al., 1990) and are responsible for high mortalities in marine bacteria (Proctor and Fuhrman, 1990). Virome analysis of planktonic microbial communities has shown a great genetic diversity (Angly et al., 2006; Hurwitz and Sullivan, 2013) and has shown that both RNA and DNA viruses are highly abundant in seawater (Culley et al., 2014). However, all of the current knowledge on viruses in seaweeds comes from studies of viruses of two small filamentous brown algae, and in particular two DNA viruses from *Ectocarpus* sp. and *Feldmania* sp. (Henry and Meints, 1992; Lanka et al., 1993). Thus the total diversity of viruses present in any seaweed has yet to be characterized, and this is particularly true for RNA viruses (as is generally the case for marine systems (Lang et al., 2009). RNA viruses are of particular interest in this context because these are the most common pathogenic viruses of terrestrial plants (Roossinck, 2012). However, research on RNA viruses in marine systems has been predominantly driven by studies on unicellular algae (Nagasaki et al., 2004; Shirai et al., 2008; Tomaru et al., 2009b, 2012).

In the present study we investigate for the first time the viral community associated with a marine red macroalga, the seaweed *Delisea pulchra*. *D. pulchra* is widespread in subtidal communities in temperate and subtropical Australia, where it can form a dominant component of the seaweed community. Interactions between the alga and its complex, surface-associated bacterial communities is mediated by chemical signals (Givskov et al., 1996; Maximilian et al., 1998; Fernandes et al., 2012; Harder et al., 2012). Environmental change can disturb this interaction resulting in bacterial-mediated disease that causes bleaching of the algal thallus (Campbell et al., 2011, 2014; Fernandes et al., 2011; Zozaya-Valdes et al., 2015). While these bacterial processes are increasingly understood, the role of viruses in the *D. pulchra* holobiont is unexplored. Here, we used transmission electron microscopy (TEM) and virome sequencing to characterize the virome component of *D. pulchra*, as a first step in understanding the interaction between the host and the viral component of its microbiome. We focus on RNA viruses because of their dominance as pathogens of macrophytes in terrestrial systems (Roossinck, 2012).

## Materials and Methods

### Collection, Processing, and Microscopy of Virome Samples

Two individuals (termed here A and B) of *D. pulchra* were collected at Malabar Beach (33°57′53.9″S 151°15′18.3″E) in Sydney at 3 m depth in January 2014 and stored in separated zip lock bags on ice until viral extraction. Each individual was washed in filter-sterilized and autoclaved seawater to remove loosely attached unspecific viral particles from the surrounding seawater. The algal tissue was then homogenized, generating samples that contained both surface-associated and intracellular viruses. Individual algae were then homogenized separately in 100 ml seaweed extraction buffer (0.1 M Tris base, 0.1 M KCl, 52 mM MgSO_4_, 0.4 M NaCl, 10 mM CaCl_2_, 0.01 M sodium sulphite, pH 7.6) in a tissue homogenizer for 5 min at 4°C. The homogenate was pressed through cheesecloth and further purified by low speed centrifugation at 2,000 × *g* for 2 min followed by a second centrifugation step at 10,000 × *g* for 10 min. The supernatant was passed through a 0.45 μm filter and viral particles were precipitated by the addition of 10% (w/v) PEG 8,000 overnight. Viral particles were pelleted by centrifugation at 7,600 × *g* for 30 min and re-suspended in VR buffer (50 mM Tris, 8 mM MgSO_4_, 0.4 M NaCl, 0.01 M sodium sulphite, pH 7.6) overnight. Cellular debris was removed by low speed centrifugation at 7,600 × *g* for 10 min. The viral containing supernatant was then further purified by density gradient ultracentrifugation. For this the viral suspension was loaded onto a 10–40% Optiprep gradient and centrifuged in a Beckman SW 41 ti swinging bucket rotor at 28,000 rpm for 2 h. After ultracentrifugation one distinct viral band was observed in both samples. These viral bands were removed separately by syringe, diluted 1:3 in VR buffer and pelleted by centrifugation at 28,000 rpm for 2 h. Viruses were re-suspended in 200 μl VR buffer without sodium sulphite. The presence of viral particles was checked by epifluorescence microscopy according to the protocol developed by Thurber et al. (2009). Viral particles were further characterized morphologically by negative staining in 2% (w/v) aqueous uranyl acetate and visualized by TEM using a FEI Technai G2 20 TEM at 200 kV and a magnification of 40,000–100,000. Images were acquired by a BM Eagle digital camera and ImageJ 1.48v (Schneider et al., 2012) was used to determine the diameter of viral particles and to improve brightness and contrast.

### Viral RNA Preparation

Two-hundred microlitres of purified and re-suspended viral particles per sample prepared as above were used for RNA extraction. Viral RNA was extracted in SDS-extraction buffer (1% SDS; 200 mM Tris; 50 mM EDTA pH 7.5) in the presence of 1% (w/v) polyvinylpyrrolidone (mol wt 40,000), 1% 2-mercaptoethanol and proteinase K (0.5 mg/ml) at 37°C for 30 min, followed by a second incubation step at 56°C for 15 min. Twice the amount of RNA-extraction buffer (150 mM Tris, 75 mM EDTA, 2.6 M NaCl, 2.6% cetyltrimethylammonium bromide (CTAB); 1% 2-mercaptoethanol; pH 7.5) was added and samples were incubated for another 15 min at 56°C. An equal volume of chloroform:isoamyl alcohol (24:1) was then added to the warm solution, mixed thoroughly and centrifuged at 12,000 × *g* at room temperature for 5 min. The chloroform:isoamyl alcohol extraction of the supernatant was repeated three times to remove contaminating polysaccharides and proteins. Finally, supernatant was transferred into a new tube and the nucleic acids were precipitated by the addition of 0.7 volume of isopropanol. After an incubation step at -20°C for 2 h nucleic acids were pelleted by centrifugation at 14,000 × *g* at 4°C for 20 min. The pellet was washed with 75% (v/v) ethanol, air dried and dissolved in 30 μl molecular-grade water. DNA was digested with the TURBO^TM^ DNAse (Life Technology) according to the manufacture’s protocol. RNA was purified by column purification using the Quick-RNA^TM^ MiniPrep kit (Zymo Research). The TruSeq stranded-RNA preparation kit (Illumina) was used for reverse transcription and cDNA library preparation. Sequencing was conducted on a MiSeq platform (Illumina) at the Ramaciotti Centre for Genomics (University of New South Wales, Sydney, NSW, Australia).

### Sequence Processing and Analysis

Sequence reads were trimmed and adaptors removed using Trimmomatic V0.32 (Bolger et al., 2014) and assembled using SPAdes 3.1.1 (Bankevich et al., 2012). Contigs were compared to the nucleotide sequence database (nt) of the National Centre for Biotechnology Information (NCBI) using blastn to identify contaminating ribosomal sequences. Contigs with an alignment length >75% of the query length and an *e*-value <10^-4^ were classified as contaminations and removed by Deconseq 0.4.3 (Schmieder and Edwards, 2011) from the sequence data. Contigs were finally classified into families using blastx and tblastx against the nr and refseq viral database (Pruitt et al., 2012) with an *e*-value cut-off smaller 10^-5^. The coverage of each contig was used to get a relative quantitative measure of the viral abundance (**Figure 3**). All partial or complete viral sequences have been submitted to the GenBank database under the accession no. LIKW00000000 (individual a) and LIKX000000000 (individual b).

### Phylogenetic Analysis

Reference sequences of the RNA-dependent RNA polymerase (RdRp) from different viruses were obtained from NCBI. Multiple alignment of amino acid sequences of the RdRp were constructed using ClustalW implemented in BioEdit 7.2.5 (Thompson et al., 1994; Hall, 1999). Phylogenetic trees were calculated based on the conserved regions of the RdRp using Mega 6.0 (Tamura et al., 2013). Phylogenetic trees were calculated using the maximum likelihood method with partial position deletion, a Poisson model and 1000 bootstrap replications. RdRp sequences analyzed in the phylogenetic tree have been submitted to GenBank database and can be found under the accession numbers *Totivirus*: KT455444-KT455461 and *Picornaviridae* KT455462-KT455464.

## Results

**FIGURE 1:**
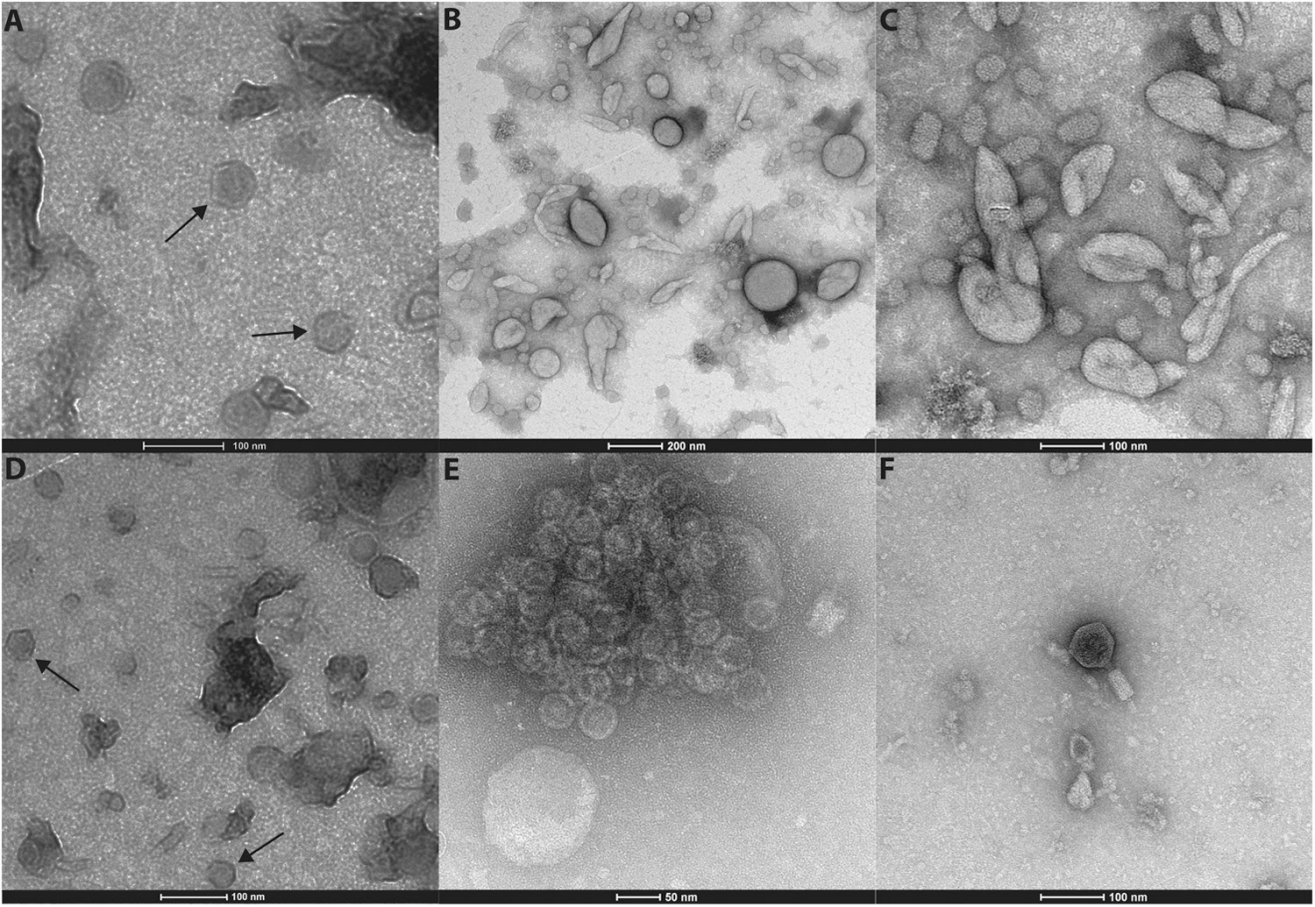
**Different morphotypes of virus like particles could be observed in both individuals.** Icosahedral viral particles with a diameter of 40 nm **(A,D)** and another size class with a diameter of 30 nm **(E)**. Bacilliform to coiled pleomorphic forms **(B,C)** were detected as well as bacteriophages **(F)**.

Transmission electron microscopy (TEM) of negative-stained virus-like particles (VLPs) revealed in both samples a range of morphotypes (**Figure 1**), including icosahedral (A, D, and E), bacilliform to coiled pleomorphic (B and C) VLPs as well as bacteriophages (F). We observed two size classes of icosahedral viral particles one with a diameter of 40 nm (**Figures 1A,D**) and one with a diameter of 30 nm (see assemblage of viral particles **Figure 1E**). The pleomorphic forms observed under TEM (**Figures 1B,C**), while virus-like, may also have been membrane vesicles originating from the homogenized algal tissue.

**FIGURE 2:**
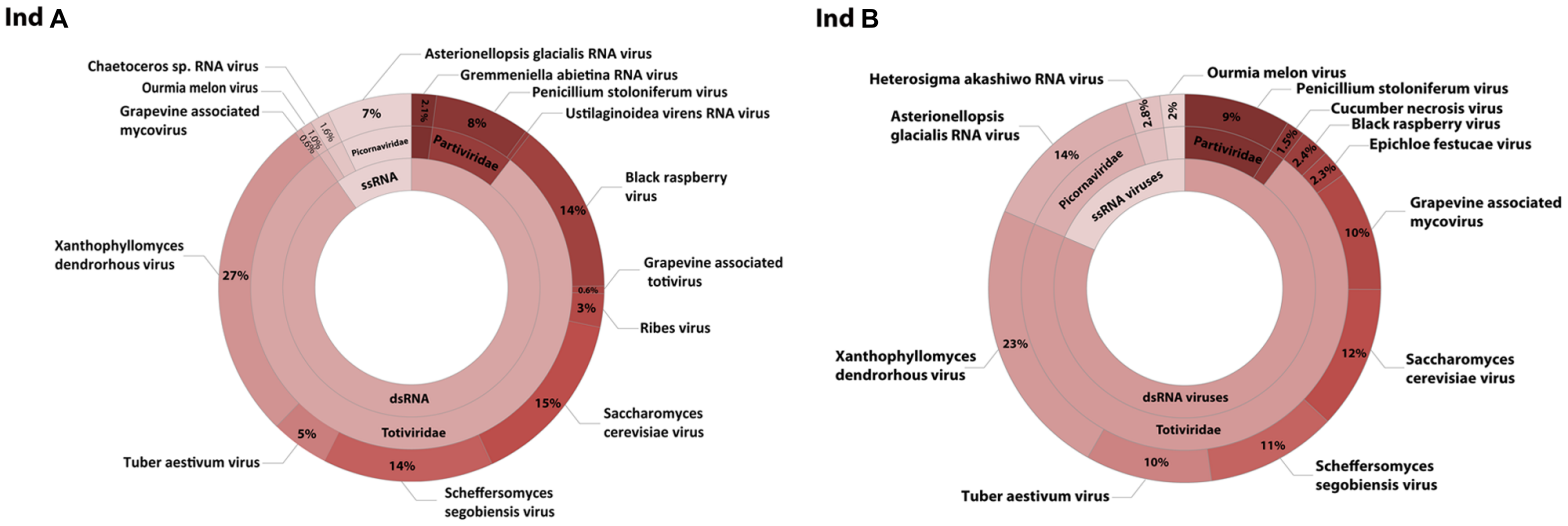
**RNA virome composition.** Taxonomic classification of viral contigs based on significant blastx and tblastx results with an *e*-value cut off of smaller than 10^-5^ from two different *D. pulchra* individuals **(A,B)**. The viral abundance is based on the coverage of each contig as a relative quantitative measure.

After removal of contaminating sequences, 267,372 reads of individual A could be assembled into 509 contigs. Based on blastx results 102 could be assigned to viruses. Individual B with 294,226 cleaned reads resulted in 369 contigs, for which 41 viral hits were found (**Table 1**). Sequence analysis revealed that both individuals contained single- and double-stranded RNA viruses. ssRNA viruses accounted for 18 and 10% of the viral community compared to 82 and 90% dsRNA viruses for individual A and B, respectively (**Figure 2**).

**Table 1:** Summary table of *Delisea pulchra* RNA viromes generated for two algal individuals A and B.

	Individual A	Individual B
Total numbers of reads	267,372	294,226
Number of contigs	509	369
Viral sequences	20%	11%
Unknown sequences	80%	89%

**FIGURE 3:**
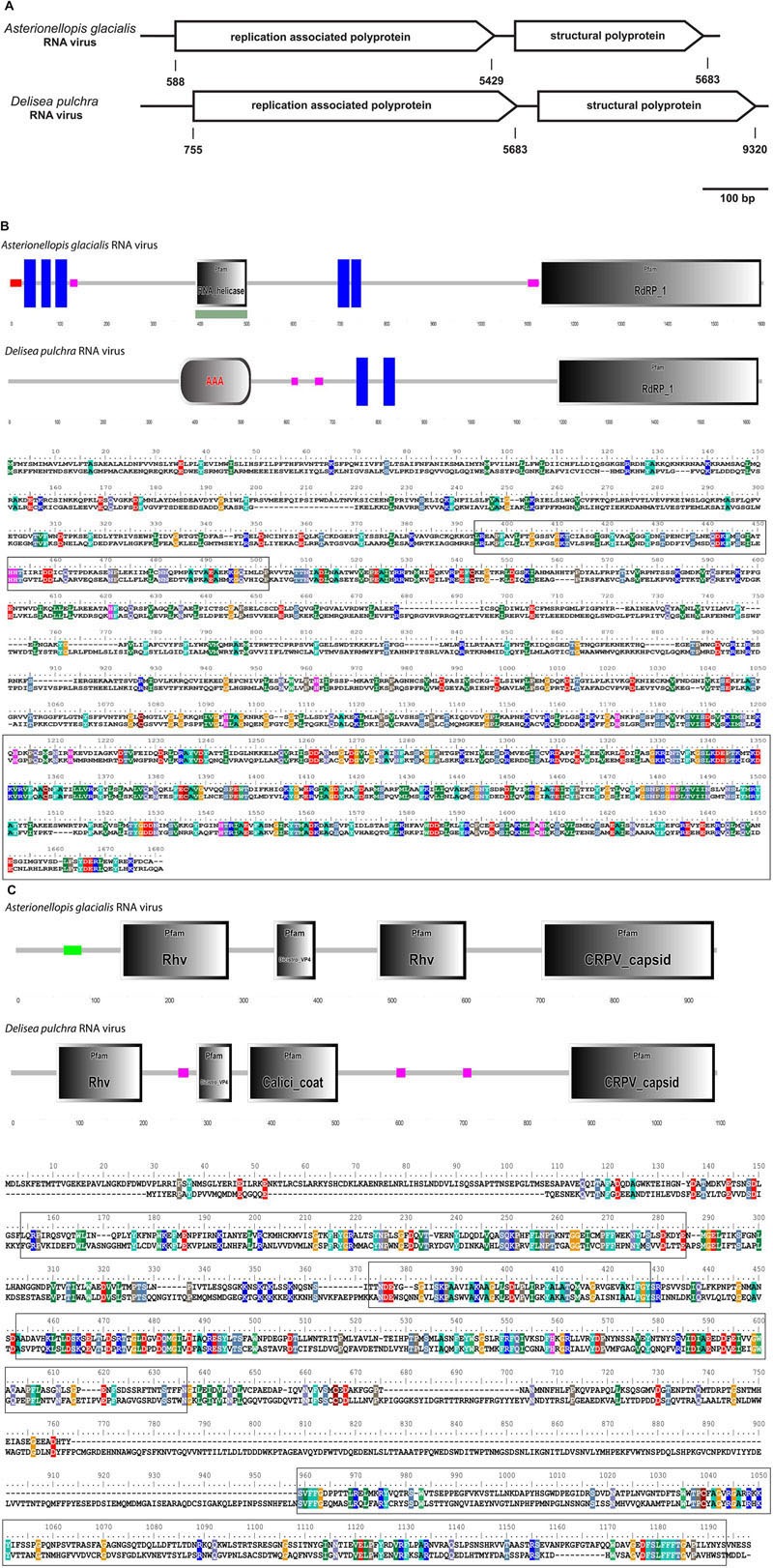
**(A–C)** Comparison between the *Asterionellopis glacialis* (“Agla”) RNA virus and the *D. pulchra* RNA virus. **(A)** The genomes of both viruses encode two polyproteins, one replication associated and one structural polyprotein. **(B)** Protein domain analysis using SMART (Simple Modular Architecture Research Tool) and sequence alignment of the replication associated polyprotein shows a similar structural organization consisting of RNA helicase or replication associated protein AAA, transmembrane proteins (blue boxes) and RdRP. Low complex regions are shown in pink. **(C)** Protein domain analysis and sequence alignment of the structural polyprotein shows four structural protein domains Rhv, DicistroVP4, Calici coat, and CRPV capsid. The Calici coat domain overlaps with the second Rhv domain of the Agla RNA virus. Sequences of conserved protein domains are highlighted in boxes.

**FIGURE 4:**
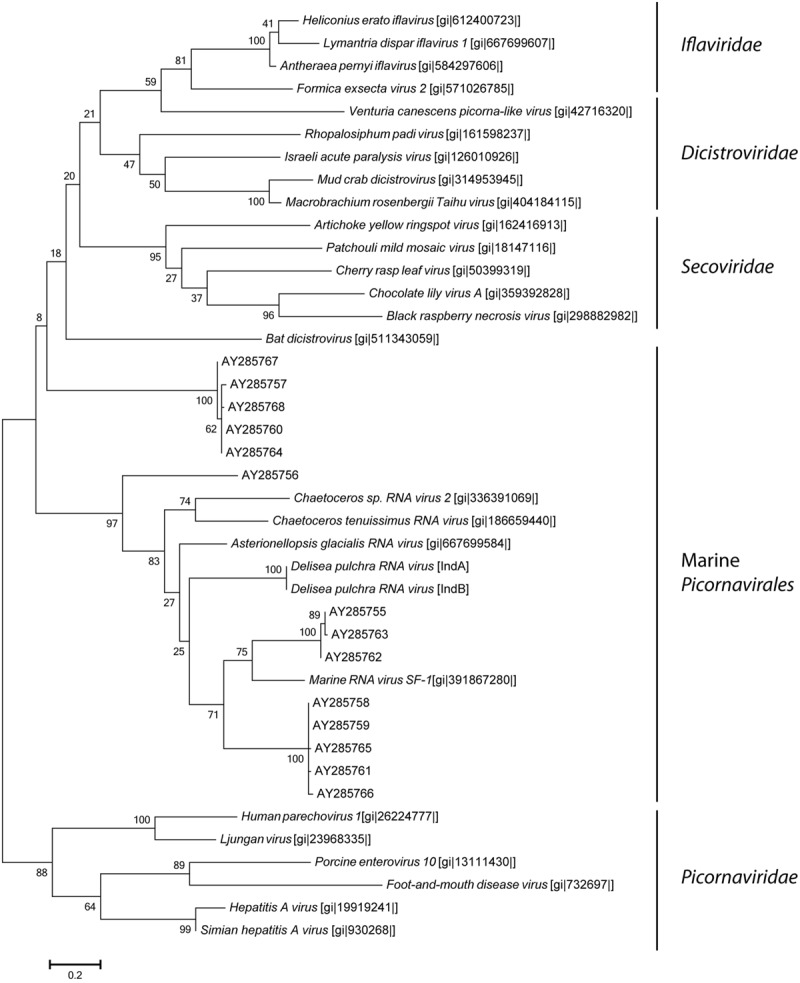
**Maximum-likelihood phylogenetic tree of *D. pulchra* associated *Picornavirales* based on the RdRp amino acid sequence compared to environmental sequences (AY285750-AY285768) of virioplankton communities of geographically diverse regions (Culley et al., 2003) and to the families *Iflaviridae, Picornaviridae, Dicistroviridae*, and *Secoviridae*.** NCBI accession numbers are shown in brackets. The scale bar represents 20% of amino acid changes between close relatives.

Most of the ssRNA viruses identified in this study showed highest sequence similarity (based on blastx results) to viruses known to infect unicellular Heterokontophyta, such as *Heterosigma akashiwo* RNA virus and *Chaetoceros* sp. RNA virus. A few viruses were closely related to viruses known to infect terrestrial plants, such as *Cucurbitaceae*. The most dominant ssRNA virus of *D. pulchra* showed highest sequence similarity to the diatom-infecting *Asterionellopsis glacialis* RNA virus (Agla RNA virus). The almost full-length genome sequence of this *D. pulchra* RNA virus with a length of 9581 bp has been submitted to GenBank under the accession number KT455464. Similar to the Agla RNA virus the *D. pulchra* RNA virus encodes two major polyproteins; one replication associated and one structural polyprotein (**Figure 3A**). Protein domain analysis via SMART (Simple Modular Architecture Research Tool; Letunic et al., 2015) revealed that the two polyproteins of both viruses are of similar architecture and amino acid composition (29 and 23% sequence similarity for the replication associated polyprotein and the structural polyprotein, respectively; **Figures 3B,C**). Within conserved domains of the polyprotein similarity reaches values of 50% for the RdRp and 46% for the structural domain Dcistrvp4. This virus could be detected in both individuals and accounted for 7–14% of the viral composition. Analysis based on the amino acid sequence of the RdRp showed that this *D. pulchra* RNA virus distinctly clusters with environmental sequences (GenBank accession numbers AY285750-AY285768) generated from geographically and seasonally diverse samples of marine virioplankton communities (Culley et al., 2003) and with other viruses isolated from marine diatoms (**Figure 4**).

**FIGURE 5:**
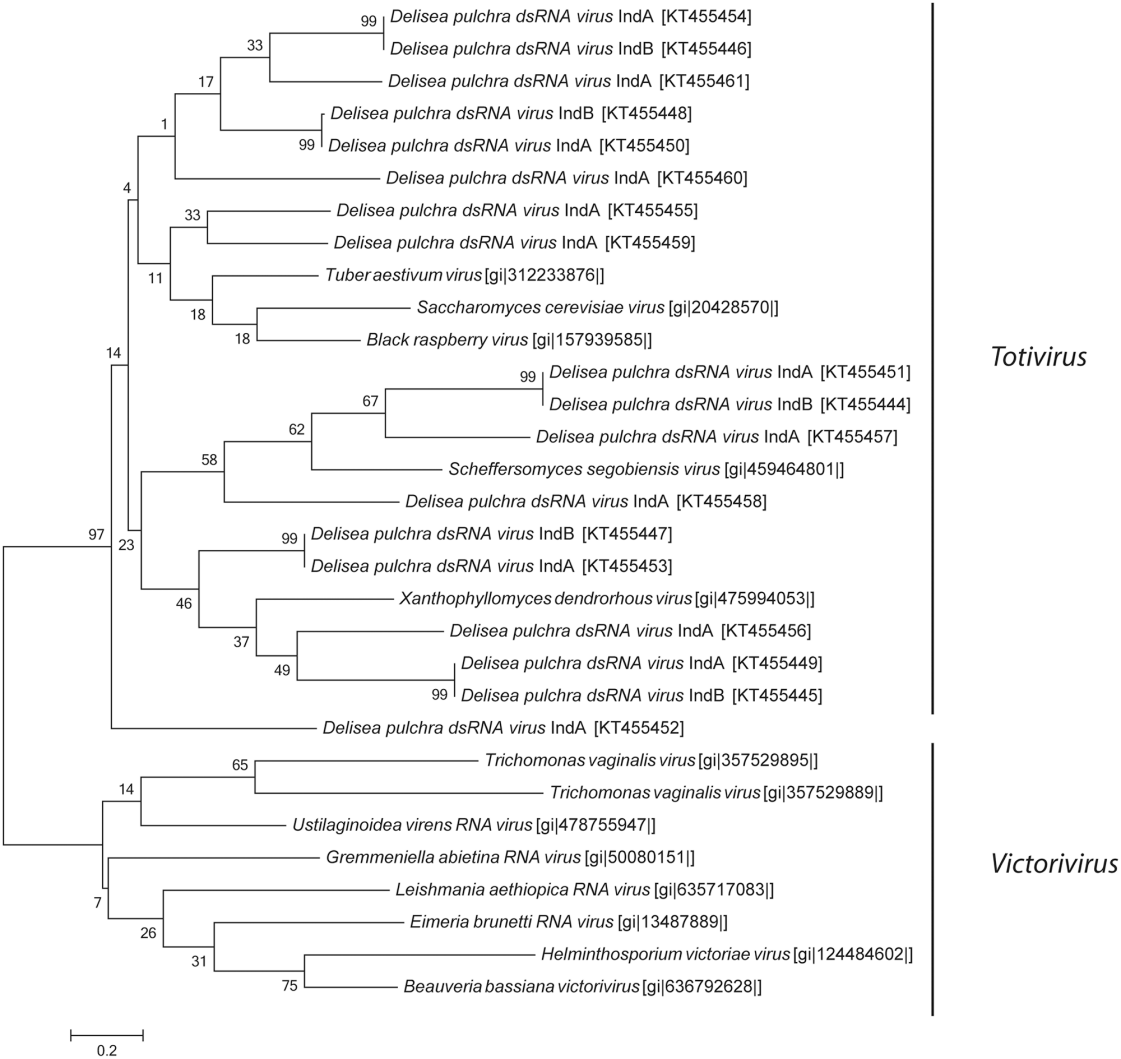
**Maximum-likelihood phylogenetic tree of *D. pulchra* associated *Totiviridae* based on the RdRp amino acid sequence compared to the genus *Victorivirus* and *Totivirus*.** NCBI accession numbers are shown in brackets. The scale bar represents 20% of amino acid changes between close relatives.

In addition, we discovered a diverse group of genetically distinct dsRNA viruses. These dsRNA viruses are taxonomically affiliated with the families *Partitiviridae* and *Totiviridae* (**Figure 2**). The relative abundance of *Partitiviridae* was comparable in both individuals and showed only a low diversity, essentially being dominated by a vius with similarity to *Penicillium stoloniferum* virus. In contrast, for the *Totiviridae* we detected more taxonomic diversity at lower levels. Comparison between the two different individuals showed that some viruses with similarity to *Xanthophyllomyces dendrorhous* virus, *Scheffersomyces segobiensis* virus, and *Saccharomyces cerevisiae* virus had relatively consistent abundances, while other viruses with similarity to *Grapevine associated* mycovirus (0.6–10%), *Black raspberry* virus (2.4–14%), and *Tuber aestivum* virus (5–10%) were more variable between both individuals. Phylogenetic analysis based on the RdRp clearly shows that *D. pulchra’s* associated *Totiviridae* form multiple, distinct clusters within the genus *Totivirus* (**Figure 5**).

Cross-tblastx comparison of *D. pulchra* viromes with other published marine RNA viromes of the virioplankton community of the Strait of Georgia, Canada (Culley et al., 2014) showed that 8–20% of the virioplankton sequences are shared with *D. pulchra* viromes based on an e-value cut-off smaller 10^-5^. The majority of shared sequences belonged to the *Picornavirales* 64–87% and 5–6% were similar to other ssRNA viruses of *D. pulchra*. 7–28% of the shared sequences of the virioplankton community showed best blast hits to unknown sequences of *D. pulchra* viromes and 0.03–2.4% to dsRNA viruses of the *Totiviridae* identified in *D. pulchra* viromes.

## Discussion

The surface of marine seaweeds are colonized by diverse bacterial communities (Lachnit et al., 2011). This association between host and microorganisms is expected to play an important role for the stability of the seaweed holobiont. In the present study we focussed on viruses associated with the red alga *D. pulchra* in order to broaden our understanding of the interaction and diversity of the seaweed holobiont, and ultimately to move toward an understanding of the role that viruses may play in disease of these seaweeds. To our knowledge, this is the first isolation of RNA viruses from seaweeds and description of entire macroalgal viromes. Because of the methods we used, these viromes include surface-associated as well as intracellular viruses, both of which may be important in the ecology of this holobiont.

There were a number of methodological challenges in extracting the virome from *D. pulchra*. During the homogenisation process of algal tissue different compounds are co-extracted along with the viruses, such as polysaccharides and secondary metabolites (Thomas and Kim, 2013) that interfere with the isolation and purification of viruses. By using different purification methods, such as filtration, low-speed centrifugation, gradient ultracentrifugation in combination with a stabilizing extraction buffer and the use of an iso-osmotic density gradient medium, we were able to isolate viruses from the marine seaweed *D. pulchra*.

The most dominant ssRNA virus detected in this study belonged to the order Picornavirales with its closest phylogenetic relation to *A. glacialis* (“Agla”) RNA virus, which infects marine diatoms and induces cell lysis (Tomaru et al., 2012). Similar to the morphology of the AglaRNA virus, we observed icosahedral viral particles by TEM. These non-enveloped viral particles were small with a diameter of 30 nm which match the typical capsid size of *Picornavirales* (Hulo et al., 2011). Together with other diatom infecting marine viruses and environmental sequences from diverse marine virioplankton viromes, this *D. pulchra* RNA virus forms a distinct cluster within the *Picornavirales*, which neighbors the plant infecting viruses of the family *Secoviridae*. Whether or not these marine RNA viruses form a new family of marine alga infecting viruses or belong to the *Secoviridae* requires further investigation and the verification of the common properties of *Secoviridae* outlined by Sanfaçon et al. (2009). In previous studies it has been shown that Picorna-like viruses are not only pathogens of animals, plants and insects in terrestrial environments, but can also be found at high abundances within the viral seawater community (Steward et al., 2013). Phylogenetic analysis based on PCR amplified RdRp sequences revealed a high phylogenetic diversity suggesting several different new families of Picorna-like viruses in the plankton viral community (Culley et al., 2003; Gustavsen et al., 2014). The isolation of the *D. pulchra* RNA virus is consistent with the broad host-range of *Picornavirales* which are known to infect terrestrial plants as well as humans (Tuthill et al., 2010) and they may have a strong impact on *D. pulchra* seaweed habitats similar to their proposed important role in structuring phytoplankton communities (Gustavsen et al., 2014).

Virome sequencing revealed that *D. pulchra* also contains a diverse group of dsRNA viruses affiliated with the genus *Totivirus*. So far, dsRNA viruses, relative to ssRNA viruses, have been underrepresented in seawater samples (Culley et al., 2006) and only a few dsRNA virus have been described from the marine plankton community, e.g., MpRNAV virus infection of the protist *Micromonas pulsilla* (Brussaard et al., 2004). Members of the *Totivirus* have a similar morphology compared to the *Picornavirales*. Viruses of the genus *Totivirus* are non-enveloped and feature an icosahedral symmetry. In contrast to *Picornavirales, Totiviruses* are larger with a diameter of 40 nm (Hulo et al., 2011). This agrees with the two different size classes of icosahedral viruses we observed by TEM. *Totiviruses* are known to infect fungi that are pathogenic to plants. The observation that *D. pulchra* is associated with viruses that are so far only known to infect fungi may point either to the presence of fungi in the holobiont *D. pulchra* or to a diverse group of marine *Totivirus* that are able to infect seaweeds. Viruses in the genus *Totivirus* predominantly infect smut fungi (Ghabrial and Suzuki, 2009). Smut fungi are also present in the marine environment and it has been demonstrated that they may cause infective disease in marine red alga (Williams et al., 2014). We could demonstrate that sequences of *Totiviruses* are also present in virioplankton communities, but compared to ssRNA viruses, particularly those of the order *Picornavirales*, these sequences are highly underrepresented. This might be explained by the transmission of *Totiviruses*, which are transmitted from cell to cell by cytoplasmatic exchange, sporogenesis or hyphal anastomosis allowing viral transmission from host to host without budding (Hulo et al., 2011).

RNA viruses represent an essential part of the marine viral community reaching or even exceeding the abundance of DNA viruses in coastal waters (Steward et al., 2013). They are the most common viral pathogens of land plants, and RNA viruses are thought to control phytoplankton blooms, although the number of studies demonstrating infection by RNA viruses of marine microorganisms is still small. For example, ssRNA viruses (*HaRNA virus*) infect the bloom forming alga *H. akashiwo* (Nagasaki et al., 1999) and HcRNA virus controls another red tide producing dinoflagellate *Heterocapsa circularisquama* (Tomaru et al., 2009a). Several ssRNA have also been shown to infect diatoms such as *Chaetoceros* (Shirai et al., 2008; Tomaru et al., 2009b, 2011) and one dsRNA has been identified in the marine environment that infects marine microalga (Brussaard et al., 2004). However, these studies represent only a snapshot of viruses compared to the high diversity of viruses that have been identified by virome sequencing of the virioplankton community. Up to now only a limited part of the virioplankton community can be linked to their eukaryotic host, such as microalgae.

We know even less about the viromes of macroalga with the lack of sequence data for macroalgal viruses in public databases significantly constraining the analysis of our data. Most of the viral sequence data in public databases are from pathogens or domesticated plants. The only viruses known from multicellular marine alga are the *Ectocarpus siliculosus* virus (Lanka et al., 1993) and *Feldmannia* sp. virus (Henry and Meints, 1992), both of which are DNA viruses. For this reason the sequence similarities of RNA viruses detected in this study represent only relatively distant relatives to known or characterized viruses (see **Figures 4** and **5**). The observed sequence similarities to viruses known to infect Heterokontophyta or diatoms therefore does not strictly speak to these organisms being the host of the viruses we have isolated, e.g., as epiphytes on *D. pulchra*. While we cannot exclude that some of the isolated viruses might be derived from surface-associated epiphytes, we took great care to select individuals that were visually free from fouling organisms and cleaned the algal surface to remove loosely attached contaminations from planktonic organisms. We thus believe that the RNA virus, found here indeed directly interacts with *D. pulchra*, which is further supported by the many taxonomic groups found consistently from both individuals. However, due to the method used in this study to isolate viruses we do not know how the viruses may be localized within the seaweed holobiont. They might be surface-associated or originate from inside the algal tissue.

*Delisea pulchra* is arguably one of the best known seaweed holobionts, but to date the focus on the microbiome of this alga has been limited to the bacterial (or archaeal) component. This microbiome is complex (Fernandes et al., 2012) and when disrupted causes a bleaching disease that significantly affects populations of this seaweed (Campbell et al., 2011, 2014; Case et al., 2011; Fernandes et al., 2012). Given the association between the viruses identified here and known plant pathogens, it is interesting to speculate that these viruses may also contribute to disease of these seaweeds. For example, temperature modulates infection of *D. pulchra* by bacteria (Case et al., 2011), and environmental conditions can also influence host-viral interactions (Larsen et al., 2008; Vega Thurber et al., 2008). Viral infection can also modulate the host derived defense, such as terpenoid synthesis and facilitate secondary infection (Luan et al., 2013; de Oliveira et al., 2015). Functional analysis of viral sequences could be one potential tool to identify such interaction, but unfortunately we did not detect any known candidate gene that might interact with the host. The only proteins that we found were replication associated or structural proteins of the isolated RNA viruses.

Moreover, in addition to the eukaryotic viruses identified by RNA analysis, we also observed bacteriophages by TEM (these were not detected in our sequencing, but RNA containing bacteriophages are less common than those containing DNA (Bollback and Huelsenbeck, 2001; Mertens, 2004). Such bacteriophages might infect bacteria on the surface of the alga, thereby changing the alga-bacterial interactions for this holobiont in unknown ways and may act as part of the holobiont immune system (Barr et al., 2013, 2014). In addition, the presence of viruses that putatively infect fungi suggests that they may control the abundance of other components of the microbiome, such as fungi. It is also interesting to draw parallels between the antibacterial compounds of *D. pulchra* (Givskov et al., 1996) and known antiviral compounds of red algae. For example, terpenes (de Oliveira et al., 2015), lectins (Takebe et al., 2013), bromophenols (Kim et al., 2011), or polysaccharides (Wang et al., 2012) have shown to possess strong antiviral activity against human pathogenic viruses. The observation in this study that marine algae are associated with a diverse viral community highlights the ecological relevance of seaweed to produce antiviral compounds. Knowing the interacting viral partners of seaweed may enable a more target-orientated drug discovery of antiviral compounds.

## Conclusion

This study shows that a large diversity of eukaryotic viruses exists in the marine seaweed *D. pulchra*. This finding argues that the interactions between the host and its associated viruses should be considered for future studies of marine holobionts. Viruses could play a key role in modulating both positive and negative interactions for the macroalgal host and be an important factor in ensuring the stability of the seaweed holobiont.

## Author Contributions

Conceived and designed the experiments: TL, TT, and PS performed the experiments: TL analyzed the data: TL Contributed reagents/materials/analysis tools:TT and PS wrote the paper: TL, TT, and PS.

## Conflict of Interest Statement

The authors declare that the research was conducted in the absence of any commercial or financial relationships that could be construed as a potential conflict of interest.
